# Systemic inflammation mediates the link between cardiometabolic multimorbidity and cognitive function among older U.S. adults: evidence from NHANES

**DOI:** 10.1097/MD.0000000000048920

**Published:** 2026-05-22

**Authors:** Zhiyuan Wang, Xuejie Hu, Yongbin Wang, Jinping Sun

**Affiliations:** a Department of Emergency Medicine, The Affiliated Hospital of Qingdao University, Qingdao, Shandong, China.

**Keywords:** cardiometabolic multimorbidity, cognitive function, inflammation, mediation analysis, NHANES

## Abstract

Existing studies suggest that cardiometabolic multimorbidity (CMM) affects cognitive function, but the role of systemic inflammation in this association remains unclear. Therefore, investigating the mechanistic role of systemic inflammation in the link between CMM and cognitive decline is crucial. This study included 2492 adults aged ≥60 years from the 2011 to 2014 National Health and Nutrition Survey. Cognitive function was assessed using 3 validated tests. Inflammatory biomarkers – systemic immune-inflammation index (SII), neutrophil-to-lymphocyte ratio (NLR), platelet-to-lymphocyte ratio, lymphocyte-to-monocyte ratio (LMR), neutrophil-to-platelet ratio (NPR), and white blood cell count – were integrated into a Comprehensive Inflammation Score (CIS) using principal component analysis. Weighted multivariate linear regression model were used to examine the associations among CMM, cognitive function, and inflammatory biomarkers. Restrictive cubic splines were used to explore the nonlinear relationship between systemic inflammation and cognitive function, and the bootstrap method was applied to examine the mediating role of systemic inflammation in the CMM–cognition relationship. Results showed that CMM was significantly associated with poorer Digit Symbol Substitution Test (DSST) performance (β = −6.57, 95% confidence interval [CI]: −8.44 to −4.70, *P* < .001), and higher systemic inflammation (NLR: β = 0.075, 95% CI: 0.040 to –0.111, *P* < .001). Systemic inflammation was also associated with poorer cognitive performance (NPR–Consortium to Establish a Registry for Alzheimer Disease: β = −2.21, 95% CI: −3.58 to −0.84, *P* = .007; LMR–Animal Fluency Test: β = 1.56, 95% CI: 0.25 to 2.87, *P* = .034; NPR**–**DSST: β = −5.93, 95% CI: −10.67 to −1.18, *P* = .017), and nonlinear associations were observed between inflammation and cognitive outcomes. Mediation analyses revealed that the SII, NLR, and LMR significantly mediated the association between CMM and DSST (SII: β = 0.053, 95% CI: 0.002 to 0.128; NLR: β = 0.133, 95% CI: 0.043 to 0.248; LMR: β = 0.118, 95% CI: 0.036 to 0.226), while NPR and CIS mediated the CMM–Consortium to Establish a Registry for Alzheimer Disease relationship (NPR: β = −0.051, 95% CI: −0.104 to −0.004; CIS: β = −0.034, 95% CI: −0.074 to −0.001). These results indicate that systemic inflammation may be a central pathway through which CMM contributes to cognitive dysfunction.

## 1. Introduction

Cardiometabolic multimorbidity (CMM) is defined as the coexistence of 2 or more cardiometabolic disorders (CMDs), including cardiovascular disease, type 2 diabetes, and stroke.^[1]^ With the global aging population on the rise, the burden of CMM has become increasingly significant. In China, the prevalence of CMM among adults over 18 years of age is approximately 5.34%.^[2]^ Substantial evidence indicates that individual cardiometabolic conditions – such as diabetes, heart disease, and stroke – are independent risk factors for cognitive decline and dementia.^[3]^ However, the synergistic detrimental effects of CMM on neurocognitive function have only recently begun to receive attention.^[1,4]^ A cohort study based on the UK Biobank revealed a significantly higher risk of dementia among individuals with CMM, along with poorer global cognitive performance.^[4]^ Furthermore, a cross-sectional analysis of 5704 older adults in rural China found associations between CMM and increased risks of Alzheimer disease (AD) and vascular dementia.^[5]^ Notably, an integrative analysis across 4 cohorts showed that a greater number of CMDs was associated with lower cognitive scores, and this association was stronger among individuals with unhealthy lifestyles.^[6]^ Collectively, these findings suggest that CMM may accelerate cognitive decline, however, the underlying mechanisms remain unclear.

Inflammation plays a central role in the progression of CMD and serves as a critical link between these conditions and cognitive impairment.^[7]^ In type 2 diabetes, inflammation exacerbates metabolic dysregulation by inducing insulin resistance^[8]^; in ischemic heart disease, circulating pro-inflammatory cytokines such as interleukin-6 and tumor necrosis factor-alpha are markedly elevated,^[9]^ while nonischemic heart disease is closely associated with NOD-like receptor family, pyrin domain containing 3 inflammasome activation.^[10]^ Poststroke neuroinflammatory cascades further contribute to secondary brain injury.^[11]^ Concurrently, systemic inflammation has been robustly linked to cognitive dysfunction in aging populations, with elevated peripheral inflammatory biomarkers correlating with hippocampal atrophy^[12]^ and microglial-driven neuroinflammation.^[13]^ Despite these insights, most studies focus on individual CMD rather than CMM,^[14]^ leaving the triadic relationship among CMM, inflammation, and cognition largely unexplored.

Derived inflammatory biomarkers based on complete blood cell counts (CBC), such as the systemic immune-inflammation index (SII), neutrophil-to-lymphocyte ratio (NLR), platelet-to-lymphocyte ratio (PLR), lymphocyte-to-monocyte ratio (LMR), and neutrophil-to-platelet ratio (NPR) have been widely adopted in the assessment of chronic inflammation due to their low cost and accessibility.^[15–18]^ Among these, the SII, which integrates neutrophil, lymphocyte, and platelet counts, was initially developed for cancer prognosis prediction^[15,16]^ and is now extensively applied in inflammation-related clinical research.^[17]^ Some researchers argue that these inflammatory biomarkers hold significant potential for application in neurological disorders, including NLR, PLR, LMR, NPR, and others^[19]^; however, the relationships between these biomarkers and neurological diseases remain incompletely understood. As mentioned earlier, few studies have examined the role of inflammation in linking CMM to cognitive function. Therefore, this study aims to examine whether inflammation mediates the relationship between CMM and cognitive function, which is helpful for us to understand the possible mechanism of cognitive impairment caused by CMM.

We used 2011 to 2014 National Health and Nutrition Survey (NHANES) data to conduct a cross-sectional analysis testing the hypothesis that inflammation mediates the CMM–cognition link. We examined CBC-derived inflammatory biomarkers – including SII, NLR, PLR, LMR, NPR, and white blood cell count (WBC) – and used principal component analysis (PCA) to create a comprehensive inflammation score (CIS) to better capture overall inflammatory burden.

## 2. Methods

### 2.1. Study design and subjects

**Figure 1.**
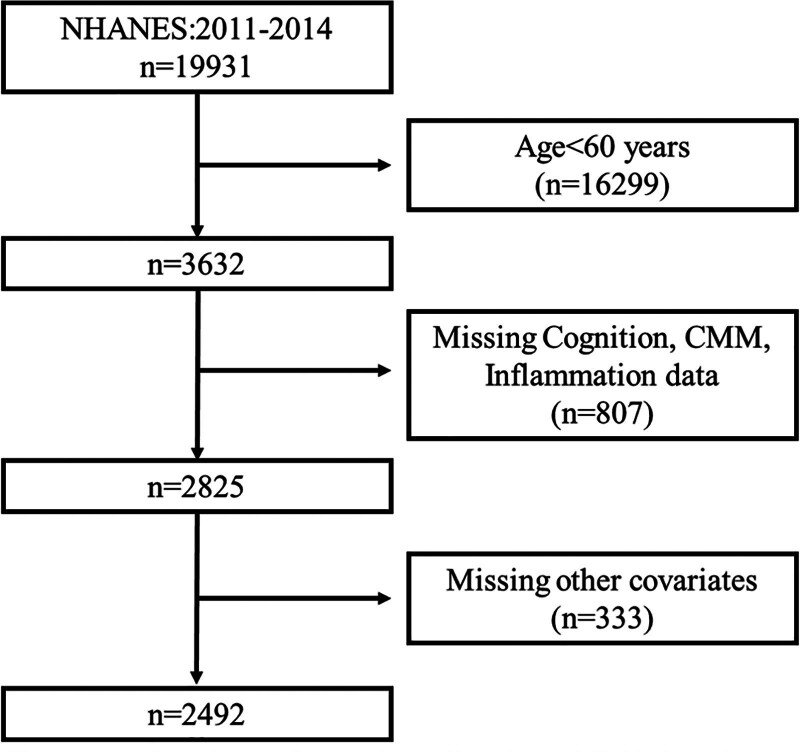
Flowchart of participants selection. CMM = cardiometabolic multimorbidity, NHANES = National Health and Nutrition Examination Survey.

The data used in this study were obtained from the NHANES, which is conducted by the National Center for Health Statistics under the Centers for Disease Control and Prevention in the United States. NHANES employs a multistage stratified sampling design to assess population health through cross-sectional surveys, with oversampling of minority ethnic groups and older adults to enhance national representativeness. All study protocols were approved by the National Center for Health Statistics Ethics Review Committee and adhered to the ethical guidelines of the Declaration of Helsinki. Written informed consent was obtained from all participants. Data collection included home interviews, telephone assessments, and standardized physical examinations, covering demographic characteristics, health behaviors, clinical measurements, and laboratory tests. The 2011 to 2014 cycles (2011–2012 and 2013–2014) were analyzed. Exclusion criteria were as follows: age < 60 years, missing cognitive function test data, incomplete blood cell count data, no history of cardiometabolic diseases (cardiovascular disease, type 2 diabetes, and stroke), and missing covariate data. A total of 2492 eligible participants were included in the final analysis, as shown in Figure 1.

### 2.2. Definition of cardiometabolic multimorbidity

The definition of CMM refers to previous studies,^[5,6]^ CMM was defined as the coexistence of ≥2 conditions from cardiovascular disease, type 2 diabetes, or stroke. Cardiovascular disease was defined as a history of congestive heart failure, coronary artery disease, angina pectoris, or myocardial infarction. Type 2 diabetes was identified based on any of the following: clinical diagnosis by a healthcare provider; insulin use; antidiabetic medication use; fasting plasma glucose level >126 mg/dL (or its equivalent); or hemoglobin A1c (HbA1c) ≥6.5%. Stroke was defined as having a prior clinical diagnosis of stroke. Participants were classified into 3 groups according to the number of coexisting conditions: no cardiometabolic disease group (No-CMD); single cardiometabolic disease group (Single-CMD); and cardiometabolic multimorbidity group (≥2 conditions, CMM).

### 2.3. Calculation of inflammatory biomarkers

This study calculated 5 inflammatory biomarkers based on data of CBC: SII = platelet count × neutrophil count/lymphocyte count; NLR = neutrophil count/lymphocyte count; PLR = platelet count/lymphocyte count; LMR = lymphocyte count/monocyte count; NPR = neutrophil count/platelet count; and the WBC uses the original data without calculation. To comprehensively assess inflammatory burden, PCA was employed to integrate SII, NLR, PLR, LMR, NPR, and WBC into a CIS.

### 2.4. Cognitive function assessment

Cognitive function was evaluated using 3 standardized tests from the Cognitive Function Questionnaire in NHANES. The Consortium to Establish a Registry for Alzheimer Disease (CERAD) assessed memory function through a multiphase task: participants learned a list of 10 unrelated words and completed 3 immediate recall trials, each scored out of 10 (total maximum 30). After other cognitive tests, a delayed recall trial required recalling the same 10 words (maximum 10 points). The total score (maximum 40) reflected short-term memory consolidation and long-term retrieval capacity. The animal fluency test (AFT) measured verbal fluency, categorical thinking, and cognitive flexibility by asking participants to list as many animal names as possible within 60 seconds; only unique, valid responses were counted. The digit symbol substitution test (DSST) evaluated processing speed and working memory: participants matched the numbers (1–9) with the corresponding symbols and filled in as many as possible within 2 minutes, earning 1 point per correct substitution. The total score directly reflected visual-motor coordination, attentional focus, and symbol-processing efficiency. Higher scores across all 3 tests indicated better cognitive performance.

### 2.5. Covariates

The covariates included in the analysis were selected from demographic and clinical data and were as follows: age (years), gender (female/male), race/ethnicity (nonHispanic White, nonHispanic Black, Mexican American, Other Hispanic, and Other race), marital status (married, divorced, separated, and other), education level (≤high school or >high school), family income-to-poverty ratio (PIR), and body mass index (BMI, kg/m^2^). Health behaviors included smoking status, defined as having smoked ≥100 cigarettes in one’s lifetime and currently smoking, and alcohol use, defined as consumption of ≥12 alcoholic drinks in the past year. Chronic diseases were defined using standardized criteria: hypertension was identified if participants met at least one of the following criteria – self-reported physician diagnosis, repeated hypertension diagnoses, antihypertensive medication use, or 2 or more systolic/diastolic blood pressure readings ≥140/90 mm Hg. Hypercholesterolemia was defined based on self-reported diagnosis, cholesterol-lowering medication use, or physician-recommended treatment. All analyses incorporated the 2-year Mobile Examination Center sampling weight (WTMEC2YR) to account for the complex survey design of NHANES.

### 2.6. Statistical analysis

Sample weights from the Mobile Examination Center interviews were adjusted to ensure national representativeness of the noninstitutionalized civilian population in the United States (weight variable: WTMEC2YR/2). Participant characteristics were presented as frequencies (n) and percentages (%) for categorical variables and as median (interquartile range [IQR]) for skewed continuous variables. PCA was performed using the prcomp function from the R (version 4.1.1; https://www.R-project.org) stats package to generate a CIS by synthesizing 6 inflammatory biomarkers: SII, NLR, PLR, NPR, LMR, and WBC. Categorical variables were compared using weighted χ^2^ tests, and continuous variables were analyzed using the Kruskal–Wallis *H* test. Weighted multivariate linear regression models were used to examine associations among CMM, cognitive function, and inflammatory biomarkers. Three models were constructed: Model 1 (unadjusted); Model 2 (adjusted for gender, age, race/ethnicity, education, marital status, BMI, and PIR); and Model 3 (Model 2 + adjustment for hypertension, hypercholesterolemia, smoking, and alcohol use). Restricted cubic splines were used to evaluate the nonlinear relationship between inflammatory biomarkers and cognitive function, and the covariate correction was consistent with that of Model 3. Mediation analysis was performed using the Process plugin based on SPSS 26.0 (IBM Corporation, Armonk, https://www.ibm.com/cn-zh/spss) with bias-corrected nonparametric Bootstrap method with 5000 resamples, 95% confidence intervals that did not include 0 were considered indicative of a significant mediation effect. All raw inflammatory biomarkers underwent base-10 logarithmic transformation (log_10_) to correct skewed distributions. Transformed data were utilized for statistical analysis, while the original variable names were used solely for descriptive purposes. All analyses were performed using the R software (version 4.1.1; https://www.R-project.org), EmpowerStats (version 2.0; http://www.empowerstats.com) and Zstats v1.0 (www.zstats.net). Statistical significance was set at *P* < .05.

## 3. Results

### 3.1. Characteristics of study participants

This study included 2492 participants aged ≥60 years, representing a weighted population of 46,935,716 individuals, as summarized in Table 1. Among them, 1203 (45.53%) were male and 1289 (54.47%) were female, with 1049 (37.93%) having ≥ 1 CMDs. The median age of the overall cohort was 68 years (IQR = 11), with the CMM group exhibiting the highest median age (70 years, IQR = 13) compared to the No-CMD group (68 years, IQR = 11) and the Single-CMD group (68 years, IQR = 11) (*P* < .001). Regarding gender distribution, the Single-CMD group had the highest proportion of males (53.72%), significantly exceeding both the No-CMD group (41.21%) and the CMM group (49.33%). Racial/ethnic composition revealed a decline in nonHispanic White participants as CMD burden increased (from 83.08% in the No-CMD group to 74.48% in the CMM group; *P* < .001), while the proportion of nonHispanic Black individuals rose significantly (from 6.45% in the No-CMD group to 11.42% in the CMM group). In terms of socioeconomic factors, the CMM group had the highest percentage of individuals with ≤12 years of education (55.37%), markedly higher than that in the Single-CMD group (42.44%) and the No-CMD group (31.81%; *P* < .001). Additionally, the CMM group had the lowest median poverty-income ratio (PIR: 2.05, IQR = 2.23), indicating greater economic disadvantage (*P* < .001). Health behaviors showed higher smoking rates in the CMM group (60.53%) compared to the Single-CMD group (56.90%) and the No-CMD group (46.49%; *P* < .001). Metabolic and disease characteristics revealed that the CMM group had the highest median BMI (30.9 kg/m^2^, IQR = 10.3), significantly exceeding other groups (*P* < .001), along with a greater number of individuals with type 2 diabetes, cardiovascular disease, and stroke compared to the Single-CMD group (*P* < .001). Additionally, the CMM group exhibited significantly higher prevalence of hypertension and hypercholesterolemia than both the Single-CMD group and the No-CMD group (*P* < .001). These findings indicate that the CMM group encounters significantly greater challenges across demographic, socioeconomic, and comorbid risk profiles.

**Table 1:** Characteristics of study participants (n = 2492).

Variables	Overall (n = 2492)	No-CMD (n = 1443)	Single-CMD (n = 771)	CMM (n = 278)	*P* value
Age (yr), median (IQR)	68 (11)	67.00 (10)	69.00 (12)	70.00 (13)	<.001***
Gender, n (%)					<.001***
Male	1203 (45.53%)	642 (41.21%)	419 (53.72%)	142 (49.33%)	
Female	1739 (54.47%)	801 (58.79%)	352 (46.28%)	136 (50.67%)	
Race, n (%)					<.001***
Non-Hispanic White	1242 (80.66%)	740 (83.08%)	365 (77.45%)	137 (74.48%)	
Non-Hispanic Black	565 (7.76%)	297 (6.45%)	196 (9.38%)	72 (11.42%)	
Mexican American	209 (3.18%)	119 (2.84%)	67 (3.60%)	23 (4.10%)	
Other Hispanic	150 (3.55%)	147 (3.40%)	78 (3.85%)	25 (3.56%)	
Other Race	226 (4.85%)	140 (4.22%)	65 (5.72%)	21 (6.43%)	
Education, n (%)					<.001***
≤High school	1182 (37.08%)	605 (31.81%)	405 (42.44%)	172 (55.37%)	
>High school	1310 (62.92%)	838 (68.19%)	366 (57.56%)	106 (44.63%)	
Marital, n (%)					.006**
Married	1378 (62.39%)	805 (63.63%)	432 (61.75%)	141 (56.22%)	
Divorced	362 (13.07%)	229 (14.16%)	95 (10.60%)	38 (13.31%)	
Separated	67 (1.19%)	33 (0.96%)	24 (1.46%)	10 (1.83%)	
Other	685 (23.36%)	376 (21.25%)	220 (26.18%)	89 (28.64%)	
BMI (kg/m^2^), median (IQR)	28.10 (7.4)	27.50 (6.8)	29.30 (7.6)	30.90 (10.3)	<.001***
PIR, median (IQR)	3.10 (3.33)	3.49 (3.09)	2.67 (3.19)	2.05 (2.23)	<.001***
Diabetes, n (%)					<.001***
No	1749 (75.53%)	1443 (100.00)	268 (42.79%)	38 (13.97%)	
Yes	753 (24.47%)	0	503 (57.21%)	240 (86.03%)	
Stroke, n (%)					<.001***
No	2319 (93.67%)	1443 (100.00)	704 (90.87%)	172 (61.05%)	
Yes	243 (6.33%)	0	67 (9.13%)	176 (38.95%)	
Heart disease, n (%)					<.001***
No	2057 (82.20%)	1443 (100.00)	570 (66.34%)	44 (13.93%)	
Yes	435 (17.80%)	0	201 (33.66%)	234 (86.07%)	
Hypertension, n (%)					<.001***
No	695 (32.08%)	494 (39.08%)	162 (21.79%)	39 (17.19%)	
Yes	1797 (67.92%)	949 (60.92%)	609 (78.21%)	239 (82.81%)	
Hypercholesterolemia, n (%)					<.001***
No	918 (36.29%)	682 (45.31%)	193 (24.07%)	43 (14.05%)	
Yes	1574 (63.71%)	761 (54.69%)	578 (75.93%)	235 (85.95%)	
Smoking, n (%)					<.001***
No	1227 (49.21%)	761 (53.51%)	350 (43.10%)	116 (39.47%)	
Yes	1265 (50.79%)	682 (46.49%)	421 (56.90%)	162 (60.53%)	
Alcohol use, n (%)					.009**
No	776 (26.95%)	431 (24.94%)	251 (30.33%)	94 (29.90%)	
Yes	1716 (73.05%)	1012 (75.06%)	520 (69.67%)	184 (70.10%)	

BMI = body mass index, CMM = cardiometabolic multimorbidity, IQR = interquartile range, No-CMD = no cardiometabolic disorders, PIR = family income-to-poverty ratio, Single-CMD = single cardiometabolic disorder.

** *P* < .01.

*** *P* < .001.

### 3.2. Inflammation and cognitive differences

Compared to the No-CMD group and the Single-CMD group, the CMM group exhibited significantly elevated levels of inflammatory markers including NLR, NPR, WBC, and CIS (all *P* < .001), whereas LMR was significantly reduced (*P* = .001). Although SII and PLR showed statistically significant differences across the 3 groups, the absolute median differences were relatively small, suggesting limited clinical relevance and warranting further investigation into their biological significance.

Regarding cognitive function, the CMM group had significantly lower scores on the CERAD, AFT, and DSST compared to the other 2 groups (all *P* < .001), indicating impaired memory function, verbal fluency, and processing speed. These findings indicate that individuals with CMM exhibit greater severity in both inflammatory burden and cognitive impairment. These findings are summarized in Table 2.

**Table 2:** Inflammation and cognitive differences among the 3 groups.

	Variables	No-CMD (n = 1443)	Single-CMD (n = 771)	CMM (n = 278)	*P* value
Inflammation, median (IQR)	SII	2.66 (0.29)	2.70 (0.31)	2.71 (0.33)	.009**
	NLR	0.32 (0.26)	0.37 (0.28)	0.40 (0.27)	<.001***
	PLR	2.09 (0.19)	2.07 (0.22)	2.07 0.24)	.010*
	LMR	0.53 (0.21)	0.51 (0.24)	0.50 (0.22)	.001**
	NPR	−1.76 (0.2)	−1.71 (0.24)	−1.66 (0.25)	<.001***
	WC	0.81 (0.15)	0.84 (0.15)	0.86 (0.14)	<.001***
	CIS	−0.3 (1.5)	−0.0 (1.8)	0.1 (1.8)	<.001***
Cognition, median (IQR)	CERAD	28.00 (9)	25.00 (8)	25.00 (9)	<.001***
	AFT	19.00 (7)	17.00 (8)	16.00 (7)	<.001***
	DSST	57.00 (22)	48.00 (21)	42.00 (24)	<.001***

AFT = animal fluency test, CERAD = Consortium to Establish a Registry for Alzheimer Disease, CIS = comprehensive inflammation score, CMM = cardiometabolic multimorbidity, DSST = digit symbol substitution test, LMR = lymphocyte-to-monocyte ratio, IQR = interquartile range, NLR = neutrophil-to-lymphocyte ratio, No-CMD = no cardiometabolic disorders, NPR = neutrophil-to-platelet ratio, PLR = platelet-to-lymphocyte ratio, SII = systemic immune-inflammation index, Single-CMD = single cardiometabolic disorders, WBC = white blood cell.

* *P* < .05.

** *P* < .01.

*** *P* < .001.

### 3.3. Associations between cardiometabolic multimorbidity and cognitive function

The associations between CMM and cognitive function are summarized in Table 3. In multivariable linear regression analyses of CERAD scores, Model 1 (unadjusted) revealed significant inverse associations for both the Single-CMD group (β = −2.17, 95% confidence interval [CI]: −2.77 to −1.58, *P* < .001) and the CMM group (β = −2.39, 95% CI: −3.77 to −1.00, *P* = .002). These associations attenuated progressively with successive covariate adjustments. In Model 2 (adjusted for sociodemographic factors), only the Single-CMD group remained significant (β = −1.09, 95% CI: −1.74 to −0.43, *P* = .004). Model 3 (further adjusted for clinical covariates) showed a weakened but still significant effect for the Single-CMD group (β = −0.99, 95% CI: −1.75 to −0.23, *P* = .022), with no significant association observed for the CMM group.

**Table 3:** The relationship between cardiometabolic multimorbidity and cognitive function.

Variables	Model 1			Model 2			Model 3		
	β	95% CI	*P* value	β	95% CI	*P* value	β	95% CI	*P* value
CERAD									
No-CMD	Ref			Ref			Ref		
Single-CMD	−2.17	−2.77 to −1.58	<.001***	−1.09	−1.74 to −0.43	.004*	−0.99	−1.75 to −0.23	.022*
CMM	−2.39	−3.77 to −1.00	.002**	−0.61	−1.73 to 0.51	.302	−0.52	−1.66 to 0.62	.383
AFT									
No-CMD	Ref			Ref			Ref		
Single-CMD	−1.68	−2.39 to −0.96	<.001***	−0.77	−1.42 to −0.11	.034*	−0.63	−1.35 to 0.08	.104
CMM	−2.52	−3.68 to −1.36	<.001***	−0.73	−1.70 to 0.25	.160	−0.62	−1.64 to 0.40	.252
DSST									
No-CMD	Ref			Ref			Ref		
Single-CMD	−7.43	−9.48 to −5.37	<.001***	−3.46	−4.86 to −2.05	<.001***	−3.22	−4.56 to −1.88	<.001***
CMM	−13.69	−16.74 to −10.65	<.001***	−6.67	−8.60 to −4.74	<.001***	−6.57	−8.44 to −4.70	<.001***

95% CI = 95% confidence interval, AFT = animal fluency test, CERAD = Consortium to Establish a Registry for Alzheimer Disease, CMM = cardiometabolic multimorbidity, DSST = digit symbol substitution test, No-CMD = no cardiometabolic disorders, Ref = reference, Single-CMD = single cardiometabolic disorder.

* *P* < .05.

** *P* < .01.

*** *P* < .001.

For AFT scores, Model 1 identified significant inverse associations for both the Single-CMD group (β = −1.68, 95% CI: −2.39 to −0.96, *P* < .001) and the CMM group (β = −2.52, 95% CI: −3.68 to −1.36, *P* < .001). However, these associations were attenuated in Model 2 (Single-CMD: β = −0.77, 95% CI: −1.42 to −0.11, *P* = .034; CMM: not significant) and became fully nonsignificant in Model 3 (*P* > .05).

In contrast, DSST score analyses exhibited robust associations. Model 1 showed strong effects for both the Single-CMD group (β = −7.43, 95% CI: −9.48 to −5.37, *P* < .001) and the CMM group (β = −13.69, 95% CI: −16.74 to −10.65, *P* < .001), which persisted in Model 2 (Single-CMD: β = −3.46; CMM: β = −6.67; both *P* < .001) and Model 3 (Single-CMD: β = −3.22; CMM: β = −6.57; both *P* < .001), although the effect sizes were attenuated. The negative association between CMM and DSST performance remained statistically significant across all models, indicating a more consistent negative impact on information processing speed.

### 3.4. Associations between cardiometabolic multimorbidity and inflammatory biomarkers

The associations between CMM and inflammatory biomarkers across different models are presented in Table 4. After adjusting for all confounders, neither the Single-CMD group nor the CMM group showed significant associations with SII or PLR. However, both the Single-CMD group (β = 0.036, 95% CI: 0.001–0.070, *P* = .042) and the CMM group (β = 0.075, 95% CI: 0.040–0.111, *P* < .001) showed significant positive associations with NLR. The CMM group (β = −0.041, 95% CI: −0.073 to −0.010, *P* = .013) showed a significant negative association with LMR, while no such relationship was observed for the Single-CMD group. Both the Single-CMD group (β = 0.036, 95% CI: 0.015–0.058, *P* = .003) and the CMM group (β = 0.079, 95% CI: 0.048–0.111, *P* < .001) revealed significant positive associations with NPR. The Single-CMD group (β = 0.020, 95% CI: 0.008–0.032, *P* = .006) showed a significant positive association with WBC, whereas no such association was observed for the CMM group. Additionally, both the Single-CMD group (β = 0.306, 95% CI: 0.091–0.520, *P* = .014) and the CMM group (β = 0.475, 95% CI: 0.189–0.762, *P* = .006) revealed significant associations with CIS.

**Table 4:** The relationship between cardiometabolic multimorbidity and inflammatory biomarkers.

Variables	Model 1			Model 2			Model 3		
	β	95% CI	*P* value	β	95% CI	*P* value	β	95% CI	*P* value
SII									
No-CMD	Ref			Ref			Ref		
Single-CMD	0.038	0.004 to 0.072	.036*	0.032	−0.005 to 0.070	.084	0.030	−0.007 to 0.068	.105
CMM	0.047	0.010 to 0.083	.017*	0.039	−0.0007 to 0.0787	.053	0.036	−0.003 to 0.076	.065
NLR									
No-CMD	Ref			Ref			Ref		
Single-CMD	0.050	0.017 to 0.083	.006**	0.036	0.003 to 0.068	.044*	0.036	0.001 to 0.070	.042*
CMM	0.090	0.058 to 0.123	<.001***	0.075	0.043 to 0.107	<.001***	0.075	0.040 to 0.111	<.001***
PLR									
No-CMD	Ref			Ref			Ref		
Single-CMD	−0.014	−0.035 to 0.007	.203	−0.0005	−0.025 to 0.015	.619	−0.0006	−0.0207 to 0.0195	.948
CMM	−0.026	−0.043 to −0.009	.005**	−0.028	−0.028 to 0.008	.292	−0.0037	−0.0255 to 0.0181	.717
LMR									
No-CMD	Ref			Ref			Ref		
Single-CMD	−0.032	−0.055 to −0.008	.013*	−0.016	−0.038 to 0.006	.167	−0.018	−0.042 to 0.006	.122
CMM	−0.052	−0.079 to −0.025	<.001***	−0.038	−0.066 to −0.010	.015	−0.041	−0.073 to −0.010	.013*
NPR									
No-CMD	Ref			Ref			Ref		
Single-CMD	0.064	0.045 to 0.083	<.001***	0.041	0.022 to 0.060	<.001***	0.036	0.015 to 0.058	.003**
CMM	0.117	0.088 to 0.146	<.001***	0.085	0.058 to 0.113	<.001***	0.079	0.048 to 0.111	<.001***
WBC									
No-CMD	Ref			Ref			Ref		
Single-CMD	0.038	0.027 to 0.049	<.001***	0.026	0.016 to 0.037	<.001***	0.020	0.008 to 0.032	.006**
CMM	0.045	0.024 to 0.066	<.001***	0.025	0.005 to 0.045	.026*	0.016	−0.004 to 0.036	.135
CIS									
No-CMD	Ref			Ref			Ref		
Single-CMD	0.393	0.159 to 0.627	.003**	0.310	0.087 to 0.533	.014*	0.306	0.091 to 0.520	.014*
CMM	0.571	0.318 to 0.823	<.001***	0.475	0.198 to 0.752	.003**	0.475	0.189 to 0.762	.006**

95% CI = 95% confidence interval, CIS = comprehensive inflammation score, CMM = cardiometabolic multimorbidity, LMR = lymphocyte-to-monocyte ratio, NLR = neutrophil-to-lymphocyte ratio, No-CMD = no cardiometabolic disorders, NPR = neutrophil-to-platelet ratio, PLR = platelet-to-lymphocyte ratio, Ref = reference, SII = systemic immune-inflammation index, Single-CMD = single cardiometabolic disorders, WBC = white blood cell.

* *P* < .05.

** *P* < .01.

*** *P* < .001.

### 3.5. Associations between inflammatory biomarkers and cognitive function

The associations between inflammatory biomarkers and cognitive function varied significantly across adjustment models (Table 5). For CERAD scores, NLR (β = −3.30, 95% CI: −4.82 to −1.80, *P* < .001), LMR (β = 4.12, 95% CI: 2.30–5.94, *P* < .001), WBC (β = −4.69, 95% CI: −7.61 to −1.77, *P* = .004), and CIS (β = −0.41, 95% CI: −0.59 to −0.23, *P* < .001) showed significant associations only in Model 1. In contrast, NPR remained significantly negatively associated with CERAD in both Model 2 (β = −2.31, 95% CI: −3.66 to −0.97, *P* = .003) and Model 3 (β = −2.21, 95% CI: −3.58 to −0.84, *P* = .007), suggesting a stronger link between NPR and memory impairment. Regarding AFT scores, NLR (β = −1.69, 95% CI: −3.04 to −0.34, *P* = .020), NPR (β = −3.26, 95% CI: −4.87 to −1.65, *P* < .001), and WBC (β = −3.32, 95% CI: −6.23 to −0.42, *P* = .032) were significant in Model 1. CIS showed significant associations in Model 1 (β = −0.27, 95% CI: −0.45 to −0.09, *P* = .007) and Model 2 (β = −0.21, 95% CI: −0.39 to −0.03, *P* = .032), while LMR demonstrated consistent associations across all models (Model 1: β = 1.75, 95% CI: 0.25–3.25, *P* = .029; Model 2: β = 1.66, 95% CI: 0.34–2.98, *P* = .023; Model 3: β = 1.56, 95% CI: 0.25–2.87, *P* = .034), indicating that LMR may serve as a key inflammatory biomarker for verbal fluency impairment. In DSST analyses, NLR (β = −7.27, 95% CI: −11.07 to −3.46, *P* < .001), PLR (β = 7.96, 95% CI: 2.53–13.39, *P* = .007), and LMR (β = 7.29, 95% CI: 1.85–12.72, *P* = .013) were significant only in Model 1. Notably, NPR exhibited robust negative associations with DSST performance across all models (Model 1: β = −16.81, 95% CI: −21.70 to −11.93, *P* < .001; Model 2: β = −6.40, 95% CI: −10.76 to −2.04, *P* = .010; Model 3: β = −5.93, 95% CI: −10.67 to −1.18, *P* = .017). Similarly, WBC remained significantly associated across all models (Model 1: β = −20.50, 95% CI: −27.24 to −13.68, *P* < .001; Model 2: β = −10.52, 95% CI: −15.71 to −5.33, *P* < .001; Model 3: β = −9.66, 95% CI: −15.19 to −4.14, *P* = .002), and CIS showed consistent associations across models (Model 1: β = −0.93, 95% CI: −1.38 to −0.49, *P* < .001; Model 2: β = −0.46, 95% CI: −0.80 to −0.11, *P* = .019; Model 3: β = −0.41, 95% CI: −0.78 to −0.04, *P* = .045). Taken together, the consistent associations of NPR, WBC, and CIS across multiple adjustment models suggest their potential role as key inflammatory biomarkers in cognitive decline related to CMM, particularly in relation to processing speed impairment.

**Table 5:** The relationship between inflammatory biomarkers and cognitive function.

Variables	Model 1			Model 2			Model 3		
	β	95% CI	*P* value	β	95% CI	*P* value	β	95% CI	*P* value
CERAD									
SII	−1.10	−2.49 to 0.29	.132	−0.43	−1.73 to 0.88	.529	−0.38	−1.69 to 0.93	.576
NLR	−3.30	−4.82 to −1.80	<.001***	−1.23	−2.67 to 0.21	.111	−1.18	−2.60 to 0.23	.123
PLR	1.63	−0.88 to 4.14	.212	0.55	−1.81 to 2.92	.651	0.51	−1.80 to 2.82	.669
LMR	4.12	2.30 to 5.94	<.001***	0.97	−1.06 to 3.00	.361	0.93	−0.98 to 2.84	.356
NPR	−6.02	−7.49 to −4.54	<.001***	−2.31	−3.66 to −0.97	.003**	−2.21	−3.58 to −0.84	.007**
WBC	−4.69	−7.61 to −1.77	.004**	−1.75	−4.43 to 0.93	.217	1.55	−4.28 to 1.19	.285
CIS	−0.41	−0.59 to −0.23	<.001***	−0.18	−0.358 to 0.003	.053	−0.17	−0.36 to 0.01	.061
AFT									
SII	−0.63	−1.93 to 0.67	.348	−0.57	−1.88 to 0.74	.405	−0.48	−1.80 to 0.85	.491
NLR	−1.69	−3.04 to −0.34	.020*	−1.18	−2.49 to 0.12	.092	−1.08	−2.41 to 0.25	.133
PLR	1.06	−0.46 to 2.58	.182	−0.39	−2.09 to 1.32	.661	−0.40	−2.14 to 1.33	.656
LMR	1.75	0.25 to 3.25	.029*	1.66	0.34 to 2.98	.023*	1.56	0.25 to 2.87	.034*
NPR	−3.26	−4.87 to −1.65	<.001***	−1.39	−2.82 to 0.03	.071	−1.23	−2.72 to 0.27	.128
WBC	−3.32	−6.23 to −0.42	.032*	−1.04	−3.51 to 1.43	.418	−0.71	−3.18 to 1.77	.583
CIS	−0.27	−0.45 to −0.09	.007**	−0.21	−0.39 to −0.03	.032*	−0.20	−0.40 to 0.01	.054
DSST									
SII	−2.78	−6.40 to 0.85	.143	−2.08	−5.03 to 0.87	.155	−1.84	−4.56 to 0.89	.206
NLR	−7.27	−11.07 to −3.46	<.001***	−1.18	−2.49 to 0.12	.092	−2.81	−5.91 to 0.29	.096
PLR	7.96	2.53 to 13.39	.007**	2.03	−2.24 to 6.30	.363	1.96	−2.31 to 6.23	.381
LMR	7.29	1.85 to 12.72	.013*	1.57	−2.62 to 5.77	.471	1.29	−2.73 to 5.31	.539
NPR	−16.81	−21.70 to −11.93	<.001***	−6.40	−10.76 to −2.04	.010*	−5.93	−10.67 to −1.18	.017*
WBC	−20.50	−27.24 to −13.68	<.001***	−10.52	−15.71 to −5.33	<.001***	−9.66	−15.19 to −4.14	.002**
CIS	−0.93	−1.38 to −0.49	<.001***	−0.46	−0.80 to −0.11	.019**	−0.41	−0.78 to −0.04	.045*

95% CI = 95% confidence interval, AFT = animal fluency test, CERAD = Consortium to Establish a Registry for Alzheimer Disease, CIS = comprehensive inflammation score, DSST = digit symbol substitution test, LMR = lymphocyte-to-monocyte ratio, NLR = neutrophil-to-lymphocyte ratio, NPR = neutrophil-to-platelet ratio, PLR = platelet-to-lymphocyte ratio, Ref = reference, SII = systemic immune-inflammation index, WBC = white blood cell.

* *P* < .05.

** *P* < .01.

*** *P* < .001.

### 3.6. Nonlinear relationship between inflammatory biomarkers and cognitive function

**Figure 2.**
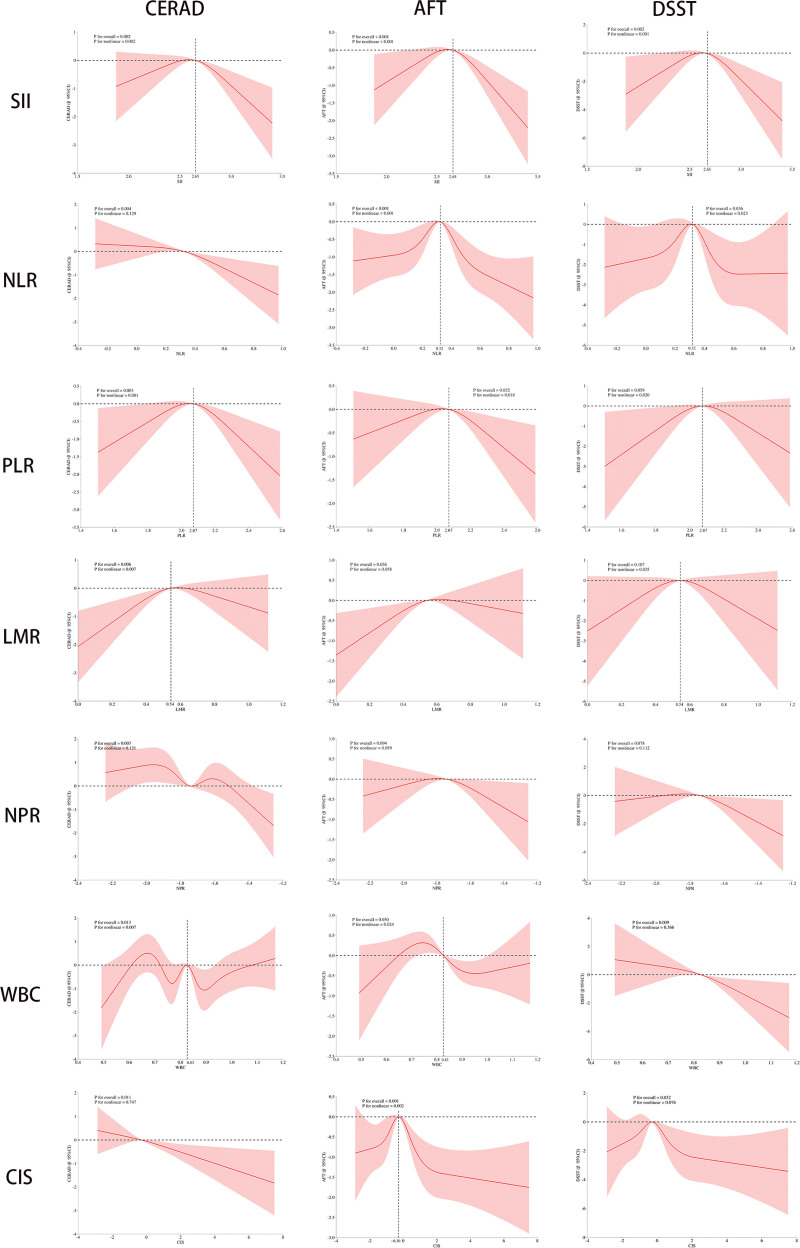
The nonlinear relationship between inflammatory biomarkers and cognitive function. AFT = animal fluency test, CERAD = Consortium to Establish a Registry for Alzheimer Disease, CIS = comprehensive inflammation score, DSST = digit symbol substitution test, LMR = lymphocyte-to-monocyte ratio, NLR = neutrophil-to-lymphocyte ratio, NPR = neutrophil-to-platelet ratio, PLR = platelet-to-lymphocyte ratio, SII = systemic immune-inflammation index, WBC = white blood cell, 95% CI = 95% confidence interval.

Nonlinear relationships between inflammatory biomarkers and cognitive function were evaluated using Restricted cubic splines models adjusted for all covariates (Fig. 2). SII exhibited significant nonlinear associations with CERAD (*P* for nonlinearity = .002), AFT (*P* for nonlinearity < .001), and DSST (*P* for nonlinearity = .001), with inflection points converging at 2.65, suggesting an accelerated risk of cognitive impairment beyond this threshold. NLR showed nonlinear associations with AFT (*P* for nonlinearity < .001) and DSST (*P* for nonlinearity = .023), with an inflection point at 0.32, but demonstrated a linear relationship with CERAD (*P* for nonlinearity = .129). PLR exhibited significant nonlinear associations across all cognitive domains – CERAD (*P* for nonlinearity = .001), AFT (*P* for nonlinearity = .018), and DSST (*P* for nonlinearity = .020) – with inflection points at 2.07. LMR displayed nonlinear associations with CERAD (*P* for nonlinearity = .007) and DSST (*P* for nonlinearity = .035), with an inflection point at 0.54, while its association with AFT approached linearity (*P* for nonlinearity = .058). NPR was the only indicator that showed linear associations across all cognitive domains (all *P* for nonlinearity > .05). WBC exhibited nonlinear relationships with CERAD (*P* for nonlinearity = .007) and AFT (*P* for nonlinearity = .024), with an inflection point at 0.83, but a linear relationship with DSST (*P* for nonlinearity = .366). CIS showed evidence of nonlinearity only with AFT (*P* for nonlinearity = .002; inflection point: −0.30). Taken together, these findings indicate that, with the exception of NPR, most inflammatory biomarkers exhibit nonlinear (predominantly inverted U-shaped) associations with cognitive performance, characterized by an increased risk of cognitive decline beyond specific inflection points.

### 3.7. The mediating effect of inflammation between cardiometabolic multimorbidity and cognitive function

**Figure 3.**
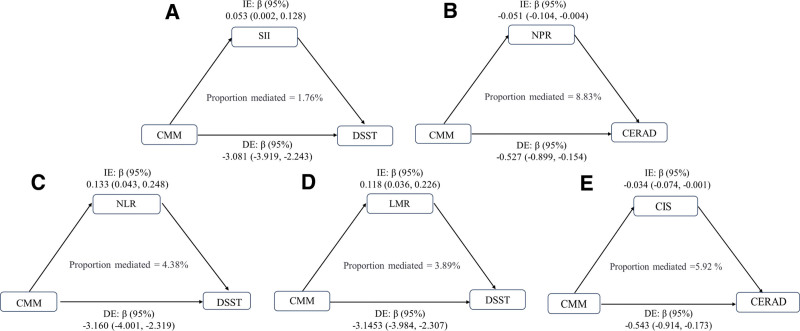
(A–E) The mediating effect of inflammation between cardiometabolic multimorbidity and cognitive function. CERAD = Consortium to Establish a Registry for Alzheimer Disease, CIS = comprehensive inflammation score, CMM = cardiometabolic multimorbidity, DE = direct effect, DSST = digit symbol substitution test, IE = indirect effect, LMR = lymphocyte-to-monocyte ratio, NLR = neutrophil-to-lymphocyte ratio, NPR = neutrophil-to-platelet ratio, SII = systemic immune-inflammation index, 95% CI = 95% confidence interval.

The mediating effect of inflammatory biomarkers in the association between CMM and cognitive function was assessed using the bootstrap method with 5000 resamples, as illustrated in Figure 3. The results revealed partial mediation by inflammation in the relationship between CMM and cognitive impairment. Specifically, in the CMM–DSST association, the proportion of the indirect effect mediated by SII in the total effect was 1.76% (Fig. 3A), whereas NPR exhibited the highest mediating effect, explaining 8.83% of the CMM–CERAD relationship (Fig. 3B). NLR and LMR mediated 4.38% and 3.89%, respectively, of the CMM–DSST association (Fig. 3C and D), and CIS mediated 5.92% of the CMM–CERAD pathway (Fig. 3E). Notably, no significant mediating effects were observed for any inflammatory biomarkers in the CMM–AFT relationship. Although the mediating effects of all inflammatory markers were relatively small (ranging from 1.76% to 8.83%), their consistent patterns across cognitive domains suggest that inflammatory pathways may represent a critical underlying mechanism in CMM-associated cognitive decline. In particular, NPR and CIS showed more pronounced mediation in memory-related domains (CERAD), whereas NLR, LMR, and SII demonstrated stronger mediating effects in processing speed domains (DSST).

## 4. Discussion

This study analyzed data from 2492 adults in the NHANES (2011–2014) to systematically examine the relationships among CMM, inflammation, and cognitive function, with CBC-derived inflammatory biomarkers serving as potential mediating variables. Results revealed that CMM was significantly associated with elevated levels of systemic inflammation and poorer cognitive performance, and that inflammation may partially mediate the adverse effects of CMM on cognition.

In the multivariable linear regression analysis examining the association between CMM and cognitive function, the detrimental impact of CMM on processing speed (DSST) remained significant after full adjustment for all confounders. This observation aligns with the established role of DSST in assessing white matter microstructural integrity,^[20,21]^ as CMD have been linked to reduced white matter volume and increased white matter hyperintensity burden.^[22]^ These findings suggest that CMM may impair white matter integrity, thereby reducing processing speed and leading to lower DSST scores. In contrast, the negative associations between CMM and memory (CERAD) or language function (AFT) yielded inconsistent results across models, with significant associations observed only in the unadjusted model. Notably, large-scale cohort studies – such as the investigation involving over 160,000 participants have demonstrated robust associations between CMM and memory decline even after covariate adjustment.^[6]^ This discrepancy might be attributable to the relatively limited sample size in the present study, which may have limited statistical power to detect subtle effects on memory and language domains. Overall, our findings are consistent with previous evidence suggesting that the impact of CMM on cognitive function may be predominantly confined to specific domains, particularly processing speed.

In weighted multivariate linear regression analyses examining the associations between CMM and inflammatory biomarkers, CMM showed a significant positive correlation with SII only in Model 1 (unadjusted). A study of U.S. adults reported that an elevated systemic inflammation response index (SIRI) was significantly associated with CMM.^[23]^ Given that both SIRI and SII are composite biomarkers of systemic inflammatory burden, these findings suggest shared pathological mechanisms underlying their associations with CMM. However, SII may be more susceptible to confounding factors. Notably, the positive association between CMM and NLR remained robust across all 3 models, aligning with prior evidence indicating that elevated NLR predicts cardiovascular mortality in patients with rheumatoid arthritis^[24]^ and independently increases the risk of cardiovascular events.^[25]^ CMM exhibited consistent negative correlations with LMR across all models. Lower LMR reflects chronic inflammation, which has been associated with increased all-cause mortality in individuals with obesity and hypertension.^[26]^ The positive association between CMM and NPR persisted after full adjustment for all covariates. The clinical relevance of NPR is particularly evident in hyperglycemic patients, where NPR levels are significantly elevated in both obstructive and nonobstructive myocardial infarction,^[27]^ and directly linked to mortality following acute myocardial infarction.^[28]^ Furthermore, after adjusting for all confounding factors, CMM remained positively correlated with CIS, further supporting its association with chronic inflammation. A UK Biobank study highlighted that chronic inflammation significantly increases the risk of CMM,^[29]^ and elevated high-sensitivity C-reactive protein has been identified as an independent correlate of CMM.^[30]^ Collectively, these findings are largely consistent with previous studies, reinforcing the strong connection between CMM and chronic inflammatory status. These results suggest that CMM may drive systemic inflammation through multiple pathways, including neutrophil activation, platelet-immune cell interactions, and monocyte/lymphocyte dysregulation, thereby exacerbating the individual’s overall inflammatory burden.

In the analysis of associations between inflammatory markers and cognitive function, NPR exhibited a significant negative correlation with CERAD across all 3 adjusted models, suggesting that elevated NPR – reflecting increased systemic inflammation – may mediate memory impairment in individuals with CMM through neutrophil-platelet interactions that promote cerebral microthrombosis.^[31]^ This finding extends the clinical relevance of NPR, which was previously primarily associated with predicting in-hospital mortality in acute myocardial infarction and after percutaneous coronary intervention.^[28,32]^ To our knowledge, this is the 1st study to reveal its potential link to cognitive dysfunction. The positive association between LMR and AFT remained robust across all 3 models, indicating that lower inflammatory states – reflected by higher LMR – may protect language function by maintaining immune homeostasis. These findings align with previous evidence showing that elevated LMR is associated with reduced risk of dementia,^[33]^ improved outcomes in patients with progressive stroke,^[34]^ and decreased incidence of postoperative cognitive dysfunction (POCD).^[35]^ This study further specifies LMR’s protective role specifically in language-related cognitive domains. Negative correlations between NPR, WBC, and CIS with DSST performance were significant across all models, reaffirming the detrimental impact of increased inflammatory burden on processing speed. Notably, CIS – a composite score integrating whole-blood cell count-derived inflammatory biomarkers – showed a unique association with DSST, potentially reflecting the heightened sensitivity of processing speed to systemic inflammation. This phenomenon has also been observed in patients with major depressive disorder,^[36]^ suggesting a cross-disease universality of inflammation-induced processing speed impairment, particularly pronounced in older populations.

We also found nonlinear associations between inflammatory biomarkers and cognitive performance, rather than simple linear relationships. In this study, except for NPR, all other indicators have nonlinear relationships with different cognitive tests. Therefore, a comprehensive discussion was conducted on the nonlinear relationship between the inflammatory state and cognitive function from an overall perspective. Specifically, when inflammatory biomarkers were below the inflection point, cognitive scores improved significantly with increasing levels of inflammation; however, once these biomarkers exceeded a certain threshold, cognitive function declined as inflammation intensified. Notably, all inflammatory biomarkers used in this study were derived from CBC, which have well-defined physiological reference ranges and reflect dynamic physiological or pathological states through numerical fluctuations.^[37]^ Within normal physiological ranges, moderate variations in these biomarkers may confer protective effects on cognitive function by modulating immune surveillance, tissue repair, and other biological processes. However, values outside the normal range – whether elevated or reduced – may indicate excessive inflammatory activation, tissue damage, or infection, potentially triggering neuroinflammation and neuronal metabolic disturbances that ultimately impair cognitive function. Furthermore, this nonlinear relationship may stem from the bidirectional regulation between the immune and nervous systems. For example, microglia and macrophages can clear cellular debris and coordinate neuronal recovery, thereby benefiting cognitive function. Conversely, their persistent activation may hinder central nervous system repair and exacerbate tissue injury, leading to worsened cognitive outcomes.^[38,39]^

The present analysis further identified that inflammation may mediate the association between CMM and cognitive impairment. Although prior research on this mediating mechanism remains limited – for instance, Liu and colleagues found no evidence of inflammatory mediation in the link between CMM and all-cause dementia among rural Chinese older adults^[5]^ – our findings offer novel supporting evidence. The Swedish SNAC-K cohort study highlighted that inflammation amplifies dementia risk in individuals with comorbid conditions,^[40]^ suggesting that synergistic interactions between inflammation and genetic factors may accelerate cognitive decline – a conclusion highly consistent with the current results. Similarly, Wang et al analysis of the NHANES database demonstrated that prolonged sleep duration (>9 hours) impairs cognitive function through inflammatory pathways.^[41]^ Together with our findings, these observations further confirm that inflammation serves as a central hub linking metabolic dysregulation, behavioral factors, and cognitive dysfunction. Moreover, lifestyle factors play an important role in modulating the relationship between CMM and cognition^[6]^: adverse behaviors such as sedentary activity and high-fat diets may indirectly impair cognitive performance by elevating inflammatory levels.^[42]^ Notably, antiinflammatory interventions may help mitigate this process. Long-term fish oil supplementation has been shown to reduce mortality risk in CMM patients through its antiinflammatory and antioxidant effects.^[43,44]^ Likewise, antiinflammatory dietary patterns demonstrate potential in lowering dementia risk and preserving brain structure – such as increasing gray matter volume and reducing white matter hyperintensities – in elderly individuals with cardiometabolic disorders.^[45]^ Collectively, these findings underscore that inflammation represents a core mechanism underlying CMM-related cognitive decline. Targeted modulation of inflammatory pathways may therefore provide a promising strategy for the prevention and management of cognitive deterioration in this population.

This study provides novel population-based evidence from NHANES (2011–2014) that systemic inflammation mediates the association between CMM and cognitive impairment, offering important insights into the biological mechanisms underlying cognitive vulnerability in individuals with CMM. Furthermore, although only peripheral inflammatory markers were used as mediating variables, the observed association still emphasizes the systemic inflammation as a mechanism bridge between CMM and cognitive decline. These results suggest that targeting inflammatory pathways – through lifestyle interventions such as antiinflammatory diets or pharmacologic immunomodulation – may represent promising strategies for mitigating cognitive risk in individuals with CMM. Future studies should adopt a longitudinal cohort design, including larger and more diverse populations, and combine classic inflammatory markers such as CRP and interleukin-6 or neuroimaging techniques to further describe the complex interaction between inflammation and cognitive function in patients with CMM. Investigations into clinically applicable antiinflammatory interventions are also warranted to inform prevention and treatment approaches for cognitive dysfunction in the context of CMM.

## 5. Conclusion

In summary, inflammation appears to be a critical mechanism linking CMM with cognitive impairment. Elevated inflammatory status were associated with poorer cognitive performance, suggesting that systemic inflammation mediates CMM-related cognitive decline. These findings highlight the potential of inflammatory biomarkers in identifying individuals at risk and underscore the importance of monitoring inflammation in CMM patients. However, given the study’s cross-sectional design, longitudinal studies are needed to confirm these results and explore whether antiinflammatory interventions can mitigate cognitive decline in this population.

## Acknowledgments

We acknowledge the CDC’s National Center for Health Statistics staff for posting the NHANES dataset online for public access.

## Author contributions

**Conceptualization:** Zhiyuan Wang, Xuejie Hu.

**Data curation:** Xuejie Hu, Yongbin Wang.

**Funding acquisition:** Jinping Sun.

**Formal analysis:** Zhiyuan Wang, Xuejie Hu.

**Supervision:** Jinping Sun, Yongbin Wang.

**Writing – original draft:** Zhiyuan Wang.

**Writing – review & editing:** Jinping Sun, Zhiyuan Wang.
